# Markers of Mineral Metabolism in Children With CKD Stages 2 to 5D

**DOI:** 10.1016/j.ekir.2026.103800

**Published:** 2026-01-24

**Authors:** Anna Tschirner, Hannah Weber, Katharina Schermuly, Ineke Böckmann, Marko Pekic, Nele Kanzelmeyer, Jens Drube, Dieter Haffner, Maren Leifheit-Nestler

**Affiliations:** 1 Department of Pediatric Kidney, Liver, Metabolic and Neurological Diseases, Pediatric Research Center, Hannover Medical School, Hannover, Germany

**Keywords:** sclerostin, FGF23, Klotho, CKD-MBD, chronic kidney disease, children

## Abstract

**Introduction:**

Changes in age- and sex-related markers for chronic kidney disease (CKD)–mineral and bone disorder (MBD) (CKD-MBD) across CKD stages 2 to 5D have not yet been studied in children.

**Methods:**

In this cross-sectional study, we investigated age, and where applicable, sex-related z-scores of 10 key markers for CKD-MBD in 170 children with stages 2-5D.

**Results:**

We identified distinct CKD stage-dependent changes in CKD-MBD markers. In CKD stage 2, elevated serum sclerostin (z-score: 0.97), total fibroblast growth factor 23 (FGF23) (z-score: 0.72) and alkaline phosphatase (AP) (z-score: 0.61) concentrations were observed in association with reduced serum phosphate (z-score: −0.62) and 1,25-dihydroxy vitamin D_3_ (1,25(OH)_2_D_3_) (z-score: −0.80) and a high prevalence of vitamin D deficiency or insufficiency (80.3%). From CKD stage 3A onward, increasingly elevated levels of intact FGF23 (iFGF23) (z-score: 0.49), and parathyroid hormone (PTH) (z-score: 1.68) were observed, as well as reduced levels of soluble Klotho (sKlotho) (z-score: −0.66). In contrast, hyperphosphatemia and hypocalcemia were only noted in patients with CKD stages 4 to 5D. CKD-MBD markers were highly associated with each other, with sclerostin being associated with total FGF23 and estimated glomerular filtration rate (eGFR). Total FGF23 was associated with serum phosphate, 25-hydroxyvitamin D3 (25(OH)D), transferrin saturation, and iFGF23.

**Conclusion:**

Elevated sclerostin, total FGF23, and AP concentrations in combination with reduced serum phosphate and (1,25(OH)_2_D_3_) as well as vitamin deficiency or insufficiency were the earliest marker for CKD-MBD in this pediatric population and were present in CKD stage 2. This preceded the parallel exponential increase in PTH and iFGF23 and reduced sKlotho with more severe CKD in children.

## Graphical abstract

**Figure:**
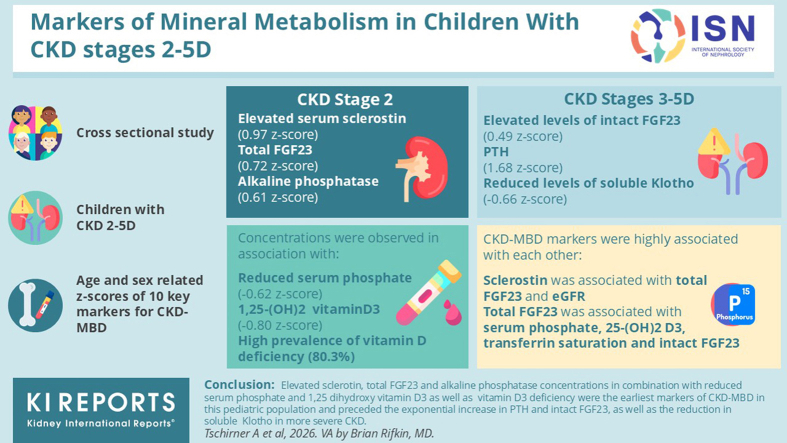

CKD is associated with complex MBD (CKD-MBD), which is defined as a triad of biochemical abnormalities (calcium [Ca], phosphate [Pi], FGF23, PTH, and vitamin D), bone abnormalities [turnover, mineralization, volume, and growth], and extrarenal calcification.^1^ The growing skeleton of children is particularly vulnerable: 29% in CKD stage 2 and > 90% on dialysis have inadequate mineralization.^2^ Moreover, CKD-MBD is associated with excessively increased cardiovascular mortality with mortality rates 100 to 500 times higher in children and young adults on dialysis treatment than in the general population.^3^, ^4^, ^5^

Studies in animal models and patients with CKD indicate that an increase in the phosphaturic hormone, FGF23, is an early event in CKD-MBD, probably due to increased Pi load per nephron perceived by the kidney and/or kidney damage leading to increased synthesis of factors that stimulate FGF23 synthesis in bone, such as glycerol-3-phosphate and lipocalin 2.^6^, ^7^, ^8^, ^9^, ^10^, ^11^, ^12^ FGF23 stimulates Pi excretion by inhibiting Pi reabsorption via binding to the FGF receptor–Klotho complex in proximal tubules resulting in downregulation of the sodium-dependent Pi cotransporters, NPT2a and NPT2c; and inhibition of the synthesis of 1,25(OH)_2_D_3_. This maintains normal Pi levels despite progressive kidney dysfunction, albeit at the expense of a decrease in 1,25(OH)_2_D_3_ concentrations, which promote hypocalcemia, compensated by increased synthesis of PTH.^6^^,^^7^ Therefore, serum Ca and Pi levels can be maintained within the normal range up to an eGFR of 30 ml/min per 1.73 m^2^, followed by overt hypocalcemia and hyperphosphatemia in CKD stage 4 to 5D. There is increasing evidence that synthesis of bone sclerostin, a Wnt-β-catenin signaling pathway inhibitor, starts to increase in early CKD, probably contributing to CKD-associated low-bone turnover and osteopenia, although robust elevations are usually noted in adults with an eGFR < 60 ml/min per 1.73 m^2^.^13^, ^14^, ^15^, ^16^

Unlike adults, children do not suffer from lifestyle- and/or age-related bone diseases, making them a unique cohort for assessing the natural course of CKD-MBD parameters as eGFR declines. We hypothesized that children with CKD already have elevated serum sclerostin levels even with mildly reduced kidney function and that the stages of CKD in which an increase in iFGF23, its biologically active form, and total FGF23 occurs differ significantly. Bones in growing children undergo continuous modeling and remodeling, and values of CKD-MBD markers vary greatly according to age and sex, which makes their assessment in this population difficult.^17^ To this aim, we first established Lambda-Mu-Sigma–based continuous reference percentiles for parameters of Pi homeostasis, allowing calculation of standardized patient z-scores.^18^^,^^19^ In this cross-sectional study, we investigated age- and, where applicable, sex-related markers for CKD-MBD in children with CKD stages 2 to 5D, including serum Pi, Ca, sclerostin, iFGF23, total FGF23, the FGF23 cofactor sKlotho, intact PTH (iPTH), 25(OH)D, 1,25(OH)_2_D_3_, and the osteoblast marker AP. We calculated the ratio of iFGF23 to Pi and Ca in serum to assess whether changes in iFGF23 were solely attributable to changes in serum Pi and Ca and severity of CKD.

## Methods

### Patients and Study Design

This cross-sectional analysis included children with CKD who participated in the prospective observational study “Growth and cognitive-motor abilities in children with nephropathic cystinosis and CKD.”^20^ Eligible children were aged between 1 and 18 years with CKD stages 2 to 5D, followed up at the pediatric nephrology outpatient clinic of the Hannover Medical School, Germany, with complete clinical data, and available blood and urine samples to assess CKD-MBD markers. Patients with renal diseases that can lead to mineral and bone metabolism disorders independently of the presence of CKD, such as tubulopathies, including nephropathic cystinosis, X-linked hypophosphatemia, Bartter syndrome, Lowe syndrome, distal renal acidosis, or primary hyperoxaluria, were excluded. All patients with CKD stages 3 to 5D regularly received dietary advice from a trained renal dietician to ensure recommended dietary requirements, that is, patients received nutritional counseling at least once a year, with the frequency being higher depending on age and CKD stage in accordance with the recommendations; for example, infants and young children undergoing dialysis treatment received 4 weekly consultations.^21^^,^^22^ A total of 170 patients with CKD enrolled in the study by December 2024 were eligible for this analysis (Table 1). Patients underwent standardized evaluations comprising medical history review, physical examination, laboratory blood tests, and collection of biosamples. The study was approved by the Institutional Review Board of Hannover Medical School (Ethic vote #6929) and was conducted in accordance with the Declaration of Helsinki. Written informed consent was obtained from all parents or legal guardians of pediatric participants, and additional patient consent or assent was secured as appropriate for the child’s age. To ensure transparent and comprehensive reporting, this manuscript adheres to the STROBE guidelines, and the completed STROBE checklist is provided in the Supplementary Material.

**Table 1:** Clinical characteristics in children with CKD stages 2 to 5D

Characteristics	All patients	CKD stage 2	CKD stage 3a	CKD stage 3b	CKD stage 4	CKD stage 5D
*n* (%)	170 (100)	68 (40)	21 (12.4)	25 (14.7)	32 (18.8)	24 (14.1)
Female, *n* (%)	65 (38.2)	35 (51.5)	6 (28.6)	8 (32)	10 (31.3)	6 (25)
Age, yrs	11.4 (7.0–15.2)	11.4^a,b^ (6.9–15.9)	9.4^a^ (6.5–12.9)	10.2^a,b^ (6.8–12.6)	11.3^a,b^ (6.3–15.2)	14.6^b^ (11.6–16.5)
Height, cm	148.7 (122.0–163.8)	152.2^a^ (129.7–167.0)	137.0^a^ (116.4–151.1)	139.0^a^ (121.5–151.5)	148.2^a^ (108.1–165.4)	157.1^a^ (134.8–169.8)
Height, z-score	−0.26^c^ (−1.27 to 0.60)	0.14^a^ (−0.92 to 1.03)	−0.29^a,b^ (−1.38 to 0.76)	−0.21^a,b^ (−1.08 to 0.22)	−0.43^a,b,d^ (−1.60 to 0.18)	−1.26^b,c^ (−2.40 to −0.18)
Body weight, kg	42.5 (23.2–57.7)	46.8^a^ (27.8–60.1)	28.6^a^ (21.1–42.0)	39.7^a^ (21.1–57.0)	38.7^a^ (18.7–56.2)	48.1^a^ (31.3–56.6)
Body weight, z-score	−0.09 (−1.10 to 0.91)	0.43^a^ (−0.72 to 1.30)	−0.64^a, b^ (−1.16 to 0.86)	0.34^a, e^ (−0.52 to 0.99)	−0.51^b, c, e^ (−1.18 to 0.11)	−1.04^b, d^ (−1.90 to 0.05)
BMI, kg/m^2^	18.0 (16.2 to 22.2)	19.5^a^ (16.5–23.6)	16.5^a^ (14.3–20.0)	21.0^a^ (16.3–22.3)	16.8^a^ (15.7–18.7)	19.3^a^ (16.1–21.5)
BMI, z-score	0.11 (−0.75 to 0.97)	0.54^a^ (−0.64 to 1.27)	0.02^a^ (−1.46 to 1.11)	0.66^a^ (0.05 to 1.22)	−0.24^a^ (−0.96 to 0.48)	−0.36^a^ (−0.98 to 0.23)
eGFR, ml/min per 1.73 m^2^	46 (24–77)	81^a^ (70–87)	51^b^ (46–57)	38^b,f^ (34–40)	24^c,f^ (21–26)	10^c,g^
UACR, mg/g	101.1 (16.7–417.2)	20.0^a^ (7.6–65.2)	91.0^a, b^ (12.1–263.4)	245.2^b^ (126.5–649.4)	375.8^b^ (160.7–1455.4)	646.5^b^ (147.8–2186.1)
Cholecalciferol, *n* (%)	104 (62.2)	28 (41.2)	13 (61.9)	20 (80)	24 (75)	19 (79.2)
Calcitriol, *n* (%)	24 (14.1)	0 (0)	0 (0)	4 (16)	7 (21.9)	13 (54.2)
Phosphate binder, *n* (%)	23 (13.5)	2 (2.9)	0 (0)	0 (0)	5 (15.6)	18 (75)
Bicarbonate, *n* (%)	39 (22.9)	0 (0)	0 (0)	8 (32)	20 (62.5)	11 (45.8)
Growth hormone, *n* (%)	17 (10)	0 (0)	0 (0)	3 (12)	7 (21.9)	7 (29.2)

BMI, body mass index; CKD, chronic kidney disease; eGFR, estimated glomerular filtration rate; UACR, urinary albumin-to-creatinine ratio.

Within-group changes: values not sharing superscript letters a, b, e, and f are significantly different from the values for other CKD stages within 1 row.

^c^ and ^d^ indicate statistical significance from healthy controls at *P* < 0.01 and *P* < 0.05, respectively.

^g^For all patients with CKD stage 5D, eGFR was set to 10 ml/m*i*n per 1.73 m^2^ because of ongoing dialysis treatment.

Data are presented as median (interquartile range) or *n* (%).

Biosamples were generally collected in the morning between 9 and 11 AM, fasting was not a requirement for sample collection. Serum, plasma, and urine samples were stored at −80 °C until measurement. Standard laboratory techniques were used to measure serum Ca, Pi, iPTH, AP, 25(OH)D, cystatin C, creatinine, hemoglobin, ferritin, transferrin; and urinary Pi, Ca, albumin, and creatinine. Bicarbonate was measured in venous blood samples by ABL 90 (Radiometer Medical, Brønshøj, Denmark). Analyses of 1,25(OH)_2_D_3_ were performed using the Liaison system (DiaSorin S.p.A., Saluggia, Italy). Enzyme-linked immunosorbent assay kits were used for quantitative determination of iFGF23 (Quidel, Catalog # 60-6600, RRID: AB_2891250), total FGF23 using the C-term FGF23 assay kit, which measures both the full-length hormone and its posttranslationally cleaved C-terminal fragments (Quidel, Catalog # 60-6100, RRID: AB_2722648), sKlotho (Immuno Biological Laboratories, Catalog # 27998, RRID: AB_2750859) in plasma, and sclerostin (TECO Medical, Catalog # TE1023-HS, RRID: AB_2894880) in serum. All assays were performed essentially as described by the manufacturers and were measured in duplicate using Tecan Infinite M200Pro (Tecan Group, Männedorf, Switzerland) and quantified with Magellan 7.2 software (Tecan Austria GmbH, Grödig, Austria). The interassay or intraassay coefficients of variation for total FGF23 were 7.6% and < 5%, for iFGF23 were 9.1% and < 5%, and for sKlotho were 3.5% and 11.4%, respectively.

eGFR was calculated using the CKID U25 formula. Whenever possible, both serum creatinine and cystatin C were included in the calculation. In cases where cystatin C was unavailable (1.2%), eGFR was calculated based on serum creatinine only. eGFR of patients undergoing dialysis was set to 10 ml/min per 1.73 m^2^. The degree of albuminuria was categorized according to the Kidney Disease: Improving Global Outcomes guidelines, with grade A1 defined as a urinary albumin-to-creatinine ratio (UACR) < 30 mg/g, grade A2 as UACR within 30 to 300 mg/g, and grade A3 as UACR > 300 mg/g.^23^^,^^24^ Tubular maximum reabsorption of Pi per GFR (TmP/GFR) was calculated using the following formula: TmP/GFR = SP − (UP/UCr) × SCr, where UP = urinary Pi, SP = serum Pi, SCr = serum Crea, and UCr = urinary Crea.^25^ Vitamin D insufficiency was defined as 25(OH)D between 50 and 75 nmol/l (20–30 ng/ml), vitamin D deficiency as 12 to 50 nmol/l (5–20 ng/ml) and severe deficiency as < 12 nmol/l (< 5 ng/ml) according to European guidelines.^26^ The proportion of patients whose iPTH levels fell within the Kidney Disease Outcomes Quality Initiative (KDOQI) recommended range was determined.^27^ Serum Ca values were adjusted for albumin.^28^ Serum Pi and Ca were classified as reduced (< −2 SD), normal (between −2 SD and 2 SD) or elevated (> 2 SD) according to reference values of healthy children.^29^

### Statistical Analysis

For anthropometric data and CKD-MBD markers, z-scores were calculated according to age and, where applicable, sex. Sex was defined as sex assigned at birth and categorized as male or female. We used local reference values for iPTH (15–65 pg/ml)^30^ and published reference values for iFGF23, sKlotho, TmP/GFR, U_Pi/Crea_, U_Ca/Crea_^18^, total FGF23, sclerostin,^19^ Pi, Ca, AP,^29^ hemoglobin,^31^ ferritin and transferrin saturation,^32^ 1,25(OH)_2_D,^33^ 25(OH)D,^34^ and the ratio of iFGF23 to Pi in serum.^35^ To calculate z-scores for the ratio of iFGF23 to Ca in serum, Lambda-Mu-Sigma–based continuous reference percentiles were generated with RefCurve 0.4.2 software^36^ using data obtained from the HAnnover Reference values for Pediatrics study as described previously.

Data are presented as median (interquartile range) or *n* (%) as appropriate. Normal distribution was tested using Kolgorov-Smirnov tests. Characteristics were analyzed using 1-sample *t* tests or Wilcoxon signed rank tests, as appropriate. Comparisons between groups were performed using 1-way analysis of variance, followed by Tukey’s multiple comparisons, or Kruskal-Wallis-test, followed by Dunn’s multiple comparisons. Simple linear and stepwise multivariable regression analyses with a threshold of *P* ≤ 0.10 were used to identify predictors of CKD-MBD markers. This included medication, example, cholecalciferol, calcitriol, bicarbonate, Pi binders, and growth hormone treatment. *P* < 0.05 was considered statistically significant. SPSS Statistics, version 29.0.2.0 (IBM Corporation, Armonk, NY) and GraphPad Prism, version 10.4.2 (GraphPad Software, Boston, MA) were used.

## Results

### Patients

This study included 170 children (38.2% female) with CKD stages 2 to 5D and a median age of 11.4 years (interquartile range: 7.0–15.2) (Table 1). The median eGFR was 46 ml/min per 1.73 m^2^; 24 patients were on dialysis (15 hemodialysis, 9 peritoneal dialysis). Underlying renal diseases comprised congenital abnormalities of the kidney and urinary tract (54.7%), glomerulopathies (20.0%), and others (25.3%). Height was significantly reduced in patients with CKD stage 4 (z-score: −0.43) and stage 5D (z-score: −1.26; each *P* < 0.05 versus healthy children), with corresponding reductions in standardized weight. The median UACR increased progressively with more severe CKD (Table 1); with 37%, 34%, and 29% of patients classified as albuminuria grades A1, A2, and A3, respectively. Medications for the treatment of CKD-MBD included cholecalciferol, calcitriol, Pi binders, sodium bicarbonate, and growth hormone in 61.2%, 14.1%, 13.5%, 22.9%, and 10.0% of cases, respectively. Detailed medication information is given in Supplementary Table S1.

### Markers of CKD-MBD According to CKD Stages

**Figure 1:**
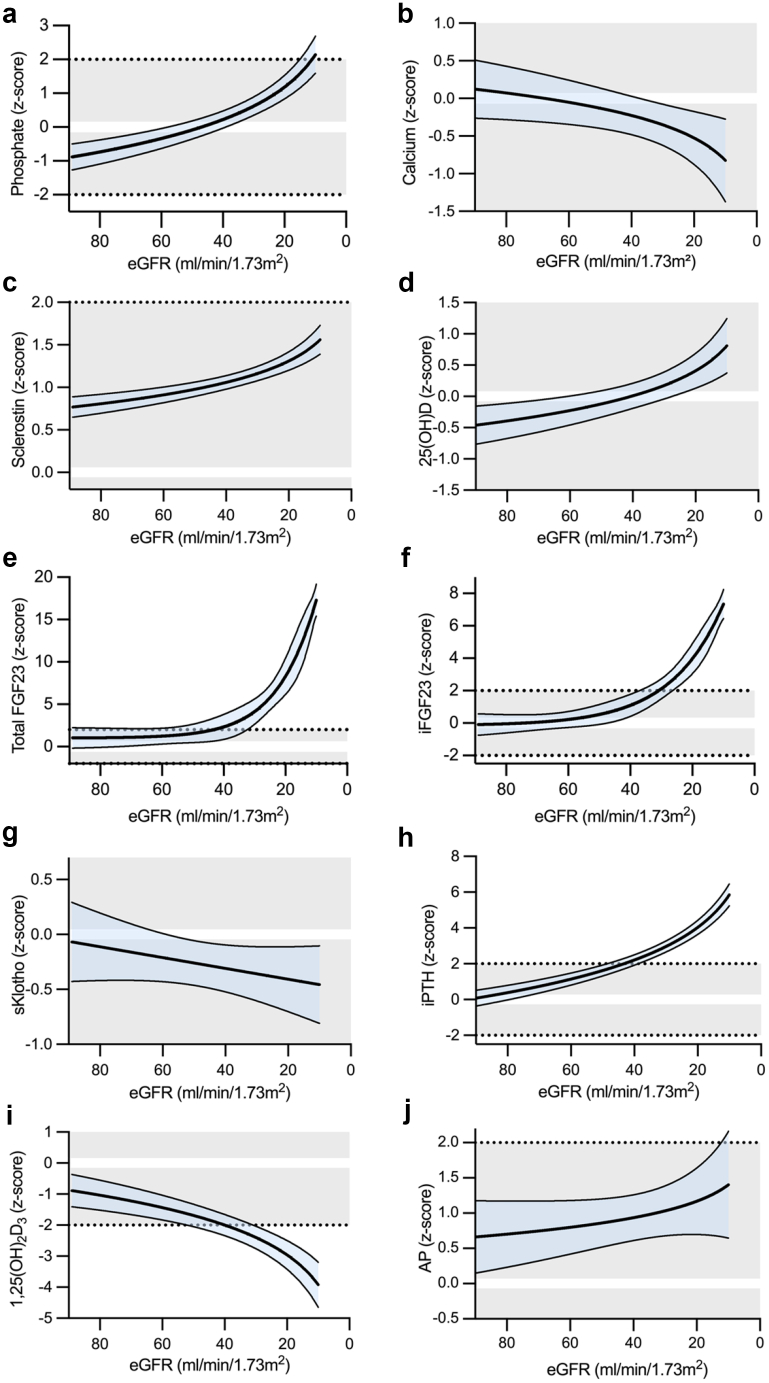
Markers for chronic kidney disease–mineral and bone disorder in children with chronic kidney disease as a function of eGFR. Values for (a) phosphate, (b) calcium, (c) sclerostin, (d) 25(OH)D, (e) total FGF23, (f) iFGF23, (g) sKlotho, (h) iPTH, (i) 1,25(OH)_2_D_3_, and (j) AP are given as age- and sex-related z-scores. For patients on dialysis treatment, eGFR was set to 10 ml/min per 1.73 m^2^. The respective best-fit function (sigmoidal, linear, or semilogarithmic) is presented, with the blue-shaded area indicating the 95% confidence interval. Gray shaded areas indicate the normal range (mean ± 2 SD), with the 0-line highlighted in white. 1,25(OH)_2_D_3_, 1,25-dihydroxy vitamin D_3_; 25(OH)D, 25-hydroxyvitamin D3; AP, alkaline phosphatase; eGFR, estimated gloemrular filtration rate; FGF23, fibroblast growth factor 23; iFGF23, intact FGF23; sKlotho, soluble Klotho; iPTH, intact parathyroid hormone.

**Figure 2:**
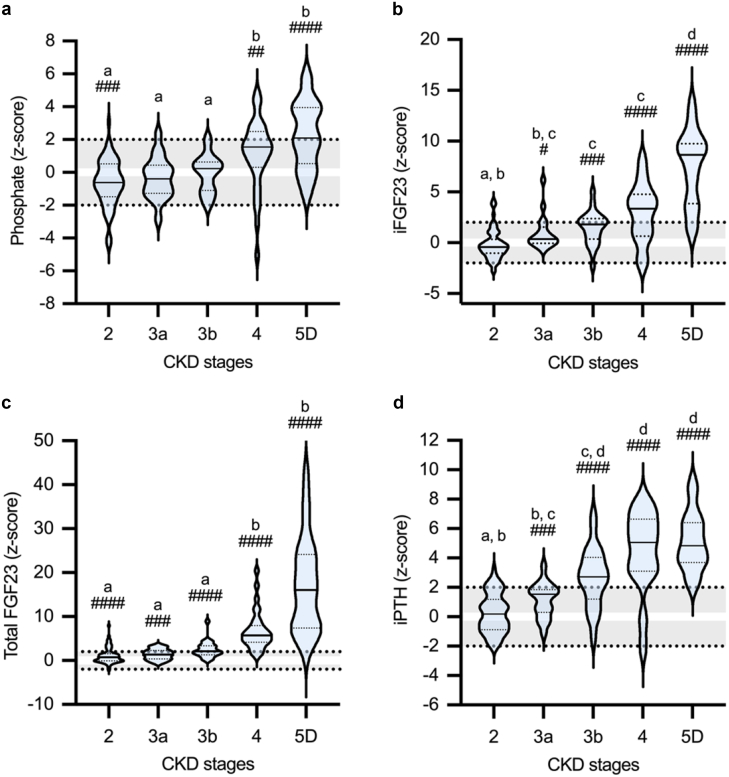
Markers for chronic kidney disease–mineral and bone disorder in children with CKD according to CKD stages. Values for (a) phosphate, (b) iFGF23, (c) total FGF23, and (d) iPTH are given as age- and sex-related z-scores. For patients on dialysis treatment, estimated glomerular filtration rate was set to 10 ml/min per 1.73 m^2^. Gray shaded areas indicate the normal range (mean ± 2 SD), with the 0-line highlighted in white. #, ##, ### and #### indicate statistical significance from healthy controls at *P* < 0.05, *P* < 0.01, *P* < 0.001, and *P* < 0.0001, respectively (1-sample *t* test or Wilcoxon signed rank test according to Kolgorow-Smirnov normality test). Values not sharing superscript letters a, b, c, and d are significantly different from the values for other CKD stages (1-way analysis of variance, followed by Tukey’s multiple comparisons, or Kruskal-Wallis-test, followed by Dunn’s multiple comparisons). CKD, chronic kidney disease; FGF23, fibroblast growth factor 23; iFGF23, intact FGF23; iPTH, intact parathyroid hormone.

Children with stage 2 CKD showed significant changes in CKD-MBD markers compared with healthy children, including elevated sclerostin (z-score: 0.77), total FGF23 (z-score: 0.72) and AP (z-score: 0.61), and reduced serum Pi (z-score: −0.62), 1,25(OH)_2_D_3_ (z-score: −0.8) and 25(OH)D (z-score: −0.78; each *P* < 0.001 vs. healthy children) (Tables 2 and 3, Figures 1 and 2, Supplementary Table S2). In contrast, iFGF23, iPTH, and sKlotho did not significantly differ from healthy children. Sclerostin levels increased further with more severe CKD peaking at CKD stage 5D (z-score: 1.86; *P* < 0.05 vs. CKD stages 2–3b; Table 3, Figure 1). Serum 25(OH)D levels were normalized with more severe CKD and more frequent cholecalciferol supplementation (*P* < 0.05, CKD stage 5D vs. CKD stage 2; Table 3; Figure 1).

**Table 2:** Parameters of mineral metabolism in children with CKD stages 2 to 5D

Characteristics	All patients	CKD stage 2	CKD stage 3a	CKD stage 3b	CKD stage 4	CKD stage 5D
Phosphate, mmol/l	1.50 (1.31–1.70)	1.37^a^ (1.20–1.52)	1.40^a, b^ (1.35–1.54)	1.51^a, b^ (1.30–1.64)	1.74^b, c^ (1.48–1.92)	1.87^c^ (1.52–2.05)
Phosphate, z-score	0.15 (−1.06 to 1.35)	−0.62^###, a^ (−1.48 to 0.52)	−0.57^a^ (−1.31 to 0.45)	0.24^a^ (−1.10 to 0.64)	1.57^##, b^ (0.41–2.62)	2.09^####, b^ (0.53–3.95)
Calcium, mmol/l	2.46 (2.38–2.53)	2.37^a^ (2.32–2.42)	2.37^a^ (2.34–2.44)	2.39^a^ (2.34–2.44)	2.32^a^ (2.26–2.44)	2.91^a^ (2.52–3.28)
Calcium, z-score	0.01 (−0.98 to 0.73)	0.07^a^ (−0.73 to 0.75)	0.01^a^ (−0.86 to 0.72)	−0.12^a^ (−0.86 to 0.73)	−0.79^#, a^ (−1.84 to 0.58)	0.37^a^ (−2.79 to 1.08)
iFGF23, pg/ml	64 (41–148)	41^a, b^ (33–54)	54^b, c^ (46–86)	80^c^ (52–95)	143^c^ (60–212)	1377^d^ (166–3197)
iFGF23, z-score	1.04^####^ (−0.39 to 3.58)	−0.43^a, b^ (−1.03 to 0.33)	0.49^#, b, c^ (−0.02 to 1.96)	1.81^###, c^ (0.37–2.39)	3.39^####, c^ (0.81–4.67)	8.66^####, d^ (3.86–9.75)
Total FGF23, RU/ml	150 (90–431)	90^a^ (68–134)	113^a^ (88–183)	145^a^ (121–186)	417^b^ (270–693)	1522^b^ (815–2186)
Total FGF23, z-score	2.19^####^ (0.70–5.74)	0.72^####, a^ (−0.07 to 1.52)	1.32^###, a^ (0.43–2.33)	2.10^####, a^ (1.29–3.38)	5.88^####, b^ (4.19–7.90)	16.07^####, b^ (7.39–24.16)
sKlotho, pg/ml	1402 (866–2122)	1535^a, b^ (868–2339)	1258^a, b^ (874–1933)	1522^a, b^ (920–3182)	1769^a^ (933–2661)	991^b^ (630–1211)
sKlotho, z-score	−0.32^#^ (−1.23 to 0.55)	−0.10^a^ (−1.04 to 0.67)	−0.66^##, a^ (−1.58 to 0.44)	−0.35^a^ (−0.83 to 1.04)	0.08^a^ (−0.58 to 0.77)	−0.96^#, a^ (−1.57 to −0.29)
iPTH, ng/l	61.9 (29.2–129.5)	31.6^a, b^ (21.3–45.5)	54.6^b, c^ (34.9–64.1)	79.9^c, d^ (45.8–130.3)	188.6^d^ (92.6–332.1)	173.2^d^ (114.3–308.1)
iPTH, z-score	2.03^####^ (−0.03 to 4.04)	0.18^a, b^ (−0.89 to 1.18)	1.68^###, b, c^ (0.46–2.12)	2.72^####, c, d^ (1.19–4.05)	5.06^####, d^ (3.12–6.61)	4.83^####, d^ (3.70–6.40)

CKD, chronic kidney disease; iFGF23, intact fibroblast growth factor 23; iPTH, intact parathyroid hormone; sKlotho, soluble Klotho; total FGF23, total fibroblast growth factor 23;

#, ##, ### and #### indicate statistical significance from healthy controls at *P* < 0.05, *P* < 0.01, *P* < 0.001, and *P* < 0.0001, respectively.

Within-group changes: values not sharing superscript letters a, b, c, and d are significantly different from the values for other CKD stages within 1 row.

Data are presented as median (interquartile range) or *n* (%).

**Table 3:** Parameters of vitamin D and bone metabolism in children with CKD stages 2 to 5D

Characteristics	All patients	CKD stage 2	CKD stage 3a	CKD stage 3b	CKD stage 4	CKD stage 5D
1,25 (OH)_2_D_3_, pmol/l	115 (89–148)	142^a^ (104–164)	114^a, b^ (89–142)	120^a, b^ (95–134)	96^b^ (73–138)	87^b^ (54–117)
1,25 (OH)_2_D_3_, z-score	−1.74^####^ (−2.93 to −0.52)	−0.80^####, a^ (−2.24 to 0.02)	−1.72^####,a, b^ (−2.92 to −0.68)	−1.47^####,a, b^ (−2.60 to −0.94)	−2.57^####, b^ (−3.90 to −0.81)	−3.07^####, b^ (−5.38 to −1.62)
25 (OH)D, nmol/l	65 (40–93)	46^a, b^ (35–73)	65^a, b, c^ (41–89)	91^c^ (67–102)	67^b, c^ (50–103)	73^c^ (55–104)
25 (OH)D, z-score	−0.01 (−0.95 to 1.00)	−0.78^###, a, b^ (−1.33 to 0.15)	−0.10^a, b, c^ (−0.94 to 0.59)	0.97^#, c^ (−0.07 to 1.46)	0.18^b, c^ (−0.59 to 1.00)	0.53^#, c^ (−0.15 to 1.77)
Sclerostin, ng/ml	0.67 (0.55–0.78)	0.63^a^ (0.54–0.72)	0.67^a^ (0.53–0.75)	0.60^a^ (0.51–0.78)	0.70^a, b^ (0.62–0.77)	1.07^b^ (0.72–1.32)
Sclerostin, z-score	1.09^####^ (0.70–1.36)	0.97^####, a^ (0.62–1.22)	1.09^####, a^ (0.62–1.29)	0.88^####, a^ (0.52–1.35)	1.17^####, a, b^ (0.92–1.35)	1.86^####, b^ (1.22–2.15)
AP, U/l	242 (167–326)	230^a^ (146–296)	241^a^ (190–284)	264^a^ (172–327)	245^a^ (160–341)	316^a^ (227–394)
AP, z-score	0.85^###^ (−0.10 to 1.90)	0.61^##, a^ (−0.20 to 1.42)	0.53^a^ (−0.71 to 1.38)	0.79^##, a^ (−0.07 to 1.32)	1.86^##, a^ (−0.18 to 2.32)	1.71^#, a^ (0.74–3.05)
iFGF23 / Pi, z-score	0.99^####^ (−0.33 to 2.64)	−0.39^, a^ (−0.99 to 0.86)	0.80^##, a, c^ (−0.16 to 1.92)	1.84^###, c^ (0.10–2.30)	2.21^####, c, d^ (0.29–3.85)	4.28^####, d^ (2.10–5.83)
iFGF23 / Ca, z-score	0.82^####^ (−0.44 to 2.74)	−0.46^a^ (−1.16 to 0.51)	0.38^##, a, b^ (−0.06 to 1.52)	1.29^###, b^ (0.27–1.91)	2.78^####, b^ (0.84–3.40)	5.19^####, c^ (3.28–5.47)

1,25(OH)_2_D_3_, 1,25-hydroxyvitamin D_3_; 25(OH)D, 25-hydroxyvitamin D; AP, alkaline phosphatase; Ca, calcium; CKD, chronic kidney disease; iFGF23, intact fibroblast growth factor 23; Pi, phosphate.

#, ##, ###, and #### indicate statistical significance from healthy controls at *P* < 0.05, *P* < 0.01, *P* < 0.001, and *P* < 0.0001, respectively.

Within-group changes: values not sharing superscript letters a, b, c, and d are significantly different from the values for other CKD stages within 1 row.

Data are presented as median (interquartile range) or *n* (%).

Total FGF23, AP, and Pi continuously increased with more severe CKD, whereas 1,25(OH)_2_D_3_ progressively decreased. Elevations in serum Pi were statistically significant at CKD stage 4 (z-score: 1.57) and 5D (z-score: 2.09, each *P* < 0.01) (Table 2, Figures 1 and 2). Serum Ca levels decreased progressively with more severe CKD and were significantly reduced in CKD stage 4 (SD: −0.79, *P* < 0.05, Table 2; Figure 1).

The increase in total FGF23 preceded the parallel exponential increase in iFGF23 and iPTH from CKD stage 3a onward. In CKD stage 5D, the concentrations of total FGF23, iFGF23, and iPTH were elevated, and the 1,25(OH)_2_D_3_ concentrations decreased by 16.07 SD, 8.66 SD, 4.83 SD, and −3.07 SD, respectively (each *P* < 0.001 vs. healthy children). Only a slight decrease in serum sKlotho was observed with more severe CKD, with significant reductions in CKD stages 3a (z-score: −0.66) and 5D (z-score: −0.96; each *P* < 0.05 vs. healthy children; Table 2, Figure 1).

**Figure 3:**
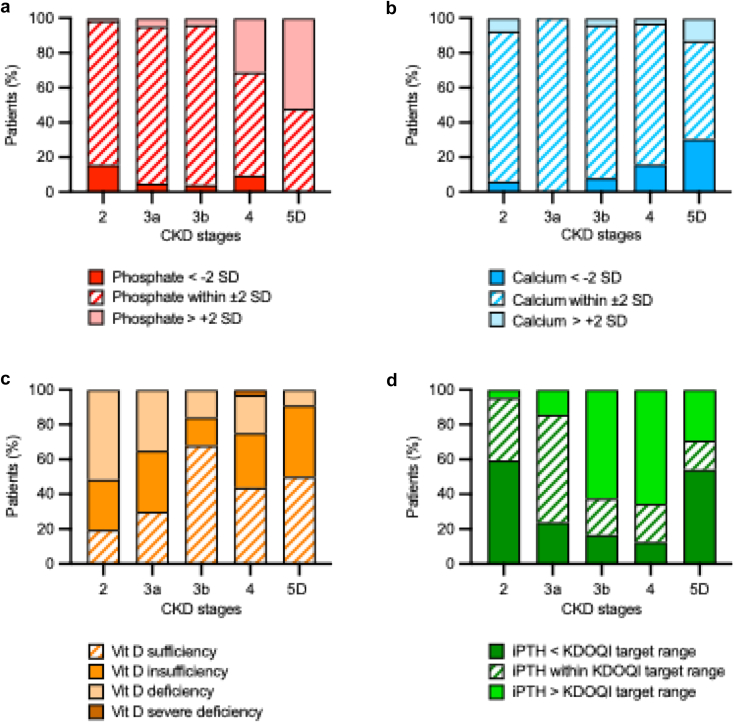
Distribution of biochemical markers of chronic kidney disease–mineral and bone disorder) in pediatric patients with CKD according to CKD stages. Data are presented as percentages of patients per CKD stage for each classification. Proportions of patients with (a) phosphate within ± 2 SD, (b) calcium within ± 2 SD, (c) Vit D status, categorized as sufficiency, insufficiency, deficiency, or severe deficiency, and (d) iPTH below, within, or above the KDOQI (Kidney Disease Outcomes Quality Initiative) target range. CKD, chronic kidney disease; iPTH, intact parathyroid hormone; KDOQI, Kidney Disease Outcomes Quality Initiative; Vit D, vitamin D.

**Figure 4:**
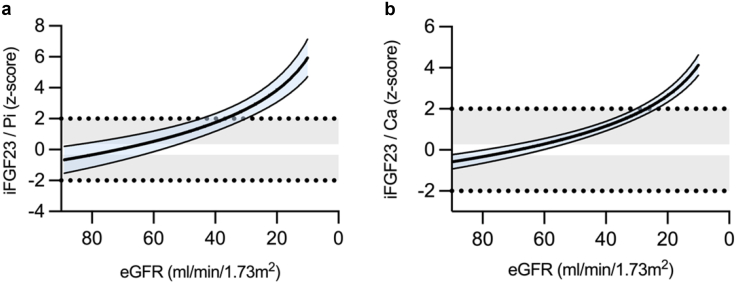
Alterations in the ratio of iFGF23 to (a) phosphate (Pi) and (b) calcium (Ca) in pediatric patients with chronic kidney disease as a function of eGFR. For patients on dialysis treatment, eGFR was set to 10 ml/min per 1.73 m^2^. Data are given as age- and sex-related z-scores. The respective best-fit function (semi-logarithmic) is presented, with the blue-shaded area indicating the 95% confidence interval. Gray shaded areas indicate the normal range (mean ± 2 SD), with the 0-line highlighted in white. eGFR, estimated gloemrular filtration rate; iFGF23, intact fibroblast growth factor 23.

The percentages of patients with normal, reduced, or elevated serum Pi and/or Ca levels, with vitamin D insufficiency or deficiency, and those with iPTH levels in the KDOQI target range are given in Figure 3. Hypophosphatemia was found in 15.6% of patients with CKD stage 2, and was rare in the other subgroups. Hyperphosphatemia occurred almost exclusively in CKD stage 4 (31.3%) and 5D (52.2%). The prevalence of hypocalcemia and hypercalcemia ranged from 0% to 30.4% and from 0% to 13.4%, respectively; and occurred more frequently in patients with CKD stage 5D (each *P* < 0.001 vs. CKD stages 2–4). In CKD stage 2 (80.3%) most patients had vitamin D deficiency or insufficiency. The prevalence of vitamin D sufficiency increased progressively across CKD stages in parallel with more frequent use of vitamin D supplements (Supplementary Table S1), peaking at CKD stage 3b (68.0%; each *P* < 0.001 vs. CKD stages 2–3a). iPTH levels were below the KDOQI target range in 59.4% of the patients with CKD stage 2, and above these targets in 62.5%, 65.6%, and 29.2% of patients with CKD stages 3b, 4, and 5D, respectively. The ratio of iFG23 to serum Pi and Ca progressively increased with more severe CKD (each *P* < 0.01 vs. healthy children) peaking at CKD stage 5 (*P* < 0.01 vs. CKD stages 2–4; Table 3, Figure 4).

### Serum Bicarbonate, Urinary Ca and Pi Excretion, Blood Hemoglobin and Serum Parameters of Iron Metabolism

Serum bicarbonate was significantly lower in patients with CKD stage 4 than those with CKD stages 2 and 5D (each *P* < 0.05) (Supplementary Table S2 and Figure S1). Median U_Ca/Crea_ was increased at all CKD stages by approximately 0.8 SD (each *P* < 0.01 vs. healthy children), whereas U_Pi/Crea_ and TmP/GFR did not differ from healthy children, with the exception of CKD stage 3b (U_Pi/Crea_ z-score: 0.12) and CKD stage 4 (TmP/GFR z-score: −0.53, each *P* < 0.05 vs. healthy children).

Because total FGF23 concentrations may be stimulated by iron deficiency in patients with CKD, we investigated blood hemoglobin and parameters of iron homeostasis (Supplementary Table S2 and Figure S1). Blood hemoglobin levels were in the normal range at CKD stages 2 and 3, and significantly reduced by −1.93 SD and −2.12 SD at CKD stage 4 and 5D, respectively (each *P* < 0.0001 vs. healthy children). Ferritin levels were in the normal range at CKD stages 2 and significantly elevated at CKD stages 3a to 5D by 0.86 SD to 1.83 SD (each *P* < 0.05 vs. healthy children). Transferrin saturation was elevated by 0.01 SD and 0.45 SD at CKD stages 3a and 3b, respectively (each *P* < 0.05 vs. healthy children).

### Predictors of CKD-MBD Markers

In the multivariable analysis, serum Pi z-score was associated with Ca (negative), and total FGF23 (positive; cumulative *r*^*2*^ = 0.524, Tables 4 and 5); Ca z-score was associated with iPTH (negative), 25(OH)D (positive), bicarbonate (positive), glomerular disease diagnosis (negative), and UACR (negative; cumulative *r*^*2*^ = 0.542); iFGF23 z-score was associated with eGFR (negative), 25(OH)D (negative), and total FGF323 (positive, cumulative *r*^*2*^ =0.657). Total FGF23 z-score was positively associated with serum Pi, 25(OH)D, and iFGF23, and negatively associated transferrin saturation (cumulative *r*^*2*^ = 0.742); sKlotho z-score was positively associated with 1,25(OH)_2_D_3_ and growth hormone treatment (positive), and negatively with diagnosis other than congenital abnormalities of the kidney and urinary tract or glomerular diseases (cumulative *r*^*2*^ = 0.204); iPTH z-score was associated with eGFR (negative), Pi (positive), Ca (negative), 1,25(OH)_2_D_3_ (positive), total FGF23 (positive), and congenital abnormalities of the kidney and urinary tract diagnosis (positive, cumulative *r*^*2*^ = 0.645). 1,25(OH)_2_D_3_ z-score was negatively associated iFGF23 (*r*^*2*^ = 0.215); 25(OH)D z-score was associated with eGFR (negative), and Ca (positive, cumulative *r*^*2*^ = 0.287); sclerostin z-score was associated with eGFR (negative) and total FGF23 (positive, cumulative *r*^*2*^ = 0.190); AP and U_Pi/Crea_ z-scores were positively associated with iPTH (*r*^*2*^ = 0.077) and Pi (*r*^*2*^ = 0.040), respectively. All other potential predictors, including cholecalciferol supplementation, calcitriol and Pi binder treatment, hemoglobin, and ferritin were no significant correlates of CKD-MBD markers.

**Table 4:** Multivariate linear regression models of variables associated with markers of bone and mineral metabolism in pediatric patients with CKD

Variable	Phosphate, z-score		Calcium, z-score		iFGF23, z-score		Total FGF23, z-score		sKlotho, z-score		iPTH, z-score	
	β (SE)	*P* value	β (SE)	*P* value	β (SE)	*P* value	β (SE)	*P* value	β (SE)	*P* value	β (SE)	*P* value
eGFR, ml/min per 1.73 m^2^	-	-	-	-	−0.301	< 0.001	-	-	-	-	−0.424	< 0.001
Phosphate, z-score			-	-	-	-	0.307	< 0.001	-	-	0.178	0.031
Calcium, z-score	−0.409	< 0.001	-	-	-	-	-	-	-	-	−0.305	< 0.001
iPTH, z-score	-	-	−0.188	0.045	-	-	-	-	-	-	-	-
1,25 (OH)_2_D_3_, z-score	-	-	-	-	-	-	-	-	0.339	< 0.001	0.182	0.014
25 (OH)D, z-score	-	-	0.393	< 0.001	−0.124	0.027	0.143	0.015			-	-
Bicarbonate, mmol/l	-	-	0.404	< 0.001	-	-	-	-	-	-		
TSAT, z-score	-	-	-	-	-	-	−0.204	< 0.001	-	-	-	-
iFGF23, z-score	-	-	-	-	-	-	0.585	< 0.001	-	-	-	-
Total FGF23, z-score	0.499	< 0.001	-	-	0.606	< 0.001	-	-	-	-	0.204	0.040
Growth hormone	-	-	-	-	-	-	-	-	0.256	< 0.001	-	-
CAKUT disease	-	-	-	-	-	-	-	-			0.207	0.003
Glomerular disease	-	-	−0.214	0.008	-	-	-	-	-	-	-	-
Other disease	-	-	-	-	-	-	-	-	−0.246	0.001	-	-
Albuminuria	-	-	−0.212	0.017	-	-	-	-	-	-	-	-
*R^2^* of the regression model		0.524		0.542		0.657		0.742		0.204		0.654

1,25(OH)_2_D_3_, 1,25-hydroxyvitamin D_3_; 25(OH)D, 25-hydroxyvitamin D; CAKUT, congenital anomalies of the kidney and urinary tract; eGFR, estimated glomerular filtration rate; iFGF23, intact fibroblast growth factor 23; iPTH, intact parathyroid hormone; *R*^*2*^, coefficient of determination; SE, standard error; sKlotho, soluble Klotho; total FGF, total fibroblast growth factor 23; TSAT, transferrin saturation; β, regression coefficient β.

**Table 5:** Multivariate linear regression models of variables associated with markers of bone and mineral metabolism in pediatric patients with CKD

Variable	1,25 (OH)_2_D_3_, z-score		25 (OH)D, z-score		Sclerostin, z-score		AP, z-score		U_Pi / Crea_, z-score	
	β (SE)	*P* value	β (SE)	*P* value	β (SE)	*P* value	β (SE)	*P* value	β (SE)	*P* value
eGFR, ml/min per 1.73 m^2^	-	-	−0.401	< 0.001	−0.232	0.015	-	-	-	-
Calcium, z-score			0.435	< 0.001	-	-	-	-	-	-
Phosphate, z-score	-	-	-	-			-	-	0.204	0.040
iPTH, z-score	-	-	-	-			0.278	0.006		
iFGF23, z-score	−0.464	< 0.001	-	-	-	-	-	-		
Total FGF23, z-score	-	-	-	-	0.252	0.008	-	-	-	-
*R^2^* of the regression model		0.215		0.287		0.190		0.077		0.042

1,25(OH)_2_D_3_, 1,25-hydroxyvitamin D_3_; AP, alkaline phosphatase; Crea, creatinine; eGFR, estimated glomerular filtration rate; iFGF23, intact fibroblast growth factor 23; iPTH, intact parathyroid hormone; Pi, phosphate; *R*^*2*^, coefficient of determination; SE, standard error; total FGF, total fibroblast growth factor 23; β, regression coefficient β.

## Discussion

**Figure 5:**
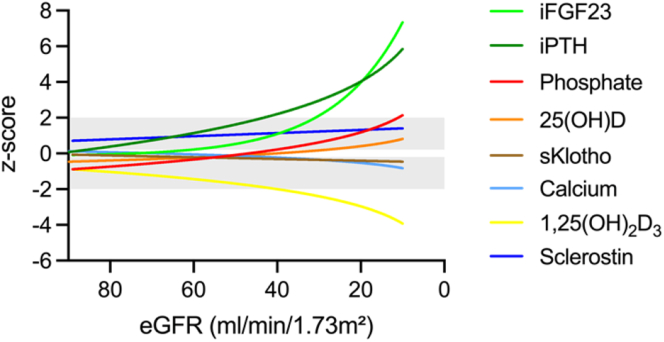
Overview of the dynamic changes of 8 key markers for chronic kidney disease– mineral and bone disorder in pediatric patients with chronic kidney disease as determined in the present study, as a function of eGFR. Values for iFGF23, iPTH, phosphate, 25(OH)D, sKlotho, calcium, 1,25(OH)_2_D_3_, and sclerostin are given as age- and sex-related z-scores. For patients undergoing dialysis, eGFR was set to 10 ml/min per 1.73 m^2^. The respective best-fit function (sigmoidal, linear, or semilogarithmic) is presented, with the blue shaded area indicating the 95% confidence interval. Gray shaded areas indicate the normal range (mean ± 2 SD), with the 0-line highlighted in white. 1,25(OH)_2_D_3_, 1,25-dihydroxy vitamin D_3_; 25(OH)D, 25-hydroxyvitamin D3; eGFR, estimated gloemrular filtration rate; iFGF23, intact fibroblast growth factor 23; iPTH, intact parathyroid hormone; sKlotho, soluble Klotho.

To our knowledge, this is the first study investigating key CKD-MBD markers in children across CKD stages 2 to 5D using adequate age, and if necessary, sex-related reference values. We found a distinct sequence in the occurrence of changes in CKD-MBD markers with more severe CKD (Figure 5). Significantly elevated sclerostin, total FGF23 and AP levels in conjunction with reduced serum Pi and 1,25(OH)_2_D_3_, and high prevalence of vitamin D deficiency or insufficiency were observed in CKD stage 2 already, compared with healthy children. This was followed in higher CKD stages by increasingly elevated iFGF23 and iPTH concentrations, whereas hyperphosphatemia and hypocalcemia as well as reduced sKlotho levels were found almost exclusively in patients with more advanced CKD. All CKD-MBD markers examined were highly associated with each other underscoring the complex pathophysiology of CKD-MBD. Our results suggest elevated sclerostin and FGF23 levels, as well as vitamin D and iron deficiency as potential targets for the treatment of CKD-MBD in children with early-stage CKD.

Elevated sclerostin concentrations in conjunction with highly prevalent vitamin D insufficiency or deficiency were already evident at CKD stage 2 and peaked in CKD stage 5D. Despite cholecalciferol supplementation in one-third of patients, vitamin D deficiency or insufficiency was found in 80.3% of children with stage 2 CKD. This appears to be higher than the results of recent population-based studies, according to which approximately 60% of healthy children and probably > 50% of the world’s population have a vitamin D deficiency or insufficiency.^37^, ^38^, ^39^

Elevated absolute sclerostin levels were previously reported in a mixed cohort of patients with stage 2 to 5 CKD before dialysis compared with healthy children, and higher levels in stage 5D CKD compared with patients with CKD before dialysis.^40^ In the latter study, circulating sclerostin levels were associated with sclerostin expression in bone, and elevated circulating and bone sclerostin levels were associated with low bone turnover as previously noted in adults with CKD stages 2 to 3.^41^ The mechanism leading to increased bone and thus circulating sclerostin in early CKD is poorly understood. In the present study, serum sclerostin was negatively associated with eGFR and positively associated with total FGF23. The latter underscores the close interaction between these bone-derived hormones and a positive feedback loop in which sclerostin stimulates osteocytes to produce more FGF23 while simultaneously influencing the effect of FGF23 on Pi and vitamin D metabolism in the context of CKD.^42^ Future studies should investigate if other known regulatory factors relevant to CKD, including TGFβ, uremic toxins, inflammatory cytokines, and reduced physical activity are associated with elevated sclerostin levels in children with early stages of CKD.^43^, ^44^, ^45^, ^46^, ^47^, ^48^

Based on animal models, an overproduction of PTH during CKD progression is thought to inhibit bone sclerostin expression.^13^^,^^43^ However, in line with a previous study in children with CKD, sclerostin levels remained high despite increasing iPTH with more severe CKD in the present study^40^; this may be at least partly related to parallel increases in total FGF23 as suggested by our multivariable analysis. Taken together, our data support the concept that elevated sclerostin levels occur early during CKD, namely already in stage 2 CKD, which seems to be independent from vitamin D deficiency and may impair adequate bone modeling and/or remodeling in these patients.^43^

The second parameter already altered in CKD stage 2 in the present study was elevated total FGF23, which was paralleled by a slight reduction in 1,25(OH)_2_D_3_ and Pi concentrations, confirming earlier studies in children and adults with CKD, which reported an eGFR threshold for total FGF23 levels of 60 to 70 ml/min per 1.73 m^2^ based on absolute values.^6^^,^^7^^,^^9^^,^^10^^,^^12^^,^^49^ Recently, an even higher threshold for total FGF23 of 102 ml/min per 1.73 m^2^ was reported in adults with preserved kidney function.^50^ Although total FGF23 level was significantly associated with serum 1,25(OH)_2_D_3_ in the univariate regression analysis as previously reported in children and adults with CKD,^7^^,^^12^^,^^50^ it was omitted in the final regression model, with serum Pi, transferrin saturation, and plasma iFGF23 appearing the only independent correlates of total FGF23. This may be at least partly related to the fact that iFGF23 was not included in the above-mentioned previous studies, which may have already addressed the associations between FGF23 and 1,25(OH)_2_D_3_ in the regression model. The association between total FGF23 levels and transferrin saturation and the finding of elevated total FGF23 levels in the face of normal iFGF23 suggest increased FGF23 cleavage because of iron deficiency. This is in line with a previous study in children with a median eGFR of 48 ml/min per 1.73 m^2^ showing significant associations between anemia and total but not with iFGF23.^51^

To our knowledge, CKD stage–dependent differences in iFGF23 levels, particularly in relation to adequate reference values, have not yet been investigated in children. We observed a parallel exponential increase in iFGF23 and iPTH with more severe CKD, with the increases becoming significant in CKD stage 3a (eGFR: 45–59 ml/min per 1.73 m^2^), accompanied by a steady further decline in 1,25(OH)_2_D_3_. This is consistent with a study in elderly adults (aged **≥** 65 years), in which eGFR thresholds for iFGF23 and iPTH were determined to be 51.6 ml/min per 1.73 m^2^ and 55.0 ml/min per 1.73 m^2^, respectively.^52^ Thus, the increase in total FGF23 in children and elderly adults with CKD clearly precedes the parallel increase in iFGF23—its biologically active form—and iPTH. Plasma iFGF23 was associated with eGFR, 25OHD, and total FGF23, whereas 1,25(OH)_2_D_3_ was omitted from the final regression model. In contrast, iPTH was associated with eGFR; serum Ca, Pi, 1,25(OH)_2_D_3_, total FGF23; and congenital abnormalities of the kidney and urinary tract disease explaining 65% of the overall variability. This suggests that the underlying mechanisms for elevated iFGF23 and iPTH levels in CKD are different, which must be considered when considering treatments to lower these levels to prevent CKD-MBD–associated comorbidities. Interestingly, in the present study, eGFR but not serum Pi was included in the final regression model for iFGF23, supporting the hypothesis that reduced renal clearance of this low molecular weight protein (molecular weight: 32 kDa) significantly contributes to elevated iFGF23 levels in patients with CKD.^53^

sKlotho, resulting from enzymatic cleavage of the extracellular domain of the membrane protein, continuously decreased with more severe CKD in the present study, which was associated with 1,25(OH)_2_D_3_ and growth hormone treatment; which is in line with a previous study in children with CKD due to nephropathic cystinosis and the fact that Klotho is mainly synthesized in the kidney and regulated by growth hormone.^35^^,^^54^, ^55^, ^56^ However, the reductions were only slight, ranging from −0.66 z-score (CKD stage 3a) to −0.96 z-score (CKD stage 5D) supporting the concept that increased iFGF23 in patients with CKD are probably not primary caused by tubular resistance to FGF23 due to Klotho deficiency.^57^

Serum AP progressively increased with mores severe CKD and correlated with iPTH suggesting increased bone turnover and/or impaired bone mineralization in our patients.^17^ Of note, AP levels were already increased in children with CKD stage 2 although iPTH levels were still within the normal range (z-score: 0.16) and even below the KDOQI PTH target range in about half of these patients. This suggests that the elevated AP values in the early stages of CKD likely reflect impaired bone mineralization, which is in line with previous bone histomorphometric studies in children with stage 2 CKD.^2^

The question arises to why 1,25(OH)_2_D_3_ concentrations were already reduced in CKD stage 2 in the face of normal iFGF23, the physiological suppressor of renal calcitriol synthesis. PTH levels , the most important inducer of renal 1,25(OH)_2_D_3_ synthesis, was below the recommended KDOQI iPTH target range in approximately half of patients with stage 2 CKD, which is likely because of the slight but significant reduction in concentrations of its stimulator, serum Pi. This could at least partially explain the reduced 1,25(OH)_2_D_3_ concentrations in patients with stage 2 CKD.

Our study has limitations. We do not have detailed information on dietary intake in our patient cohort, for example, in the months prior to the visit. Therefore, the observed CKD stage–dependent differences in CKD-MBD biomarkers may be at least partly attributable to differences in Ca and/or Pi intake. However, all patients with CKD stages 3 to 5D regularly received dietary advice by a trained renal dietician to ensure recommended dietary requirements.^21^^,^^22^ Blood samples were not strictly taken in the fasting state, which may have biased the results of our study. We did not investigate the bone expression of the bone–derived CKD-MBD parameters but measured their circulating levels, for example, FGF23 and sclerostin. However, previous studies in children and adult CKD patients have shown a good correlation between circulating bone markers and bone expression.^40^, ^41^, ^42^, ^43^^,^^58^ Finally, the relatively small number of subjects may have made it impossible to observe a correlation between cholecalciferol supplementation and 25OHD levels in our study.

In conclusion, elevated sclerostin, total FGF23, and AP levels in conjunction with reduced serum Pi and 1,25(OH)_2_D_3_ and high prevalence of vitamin D deficiency or insufficiency were already noted in CKD stage 2 in this pediatric population, suggesting that increased sclerostin already may impair bone modeling and/or remodeling in early CKD. This was followed in patients with CKD stage 3a by increasingly elevated iFGF23 and iPTH concentrations, while hyperphosphatemia and hypocalcemia as well as reduced sKlotho levels were found almost exclusively in patients with more advanced CKD. Thus, the compensatory mechanisms of elevated iFGF23 and iPTH appear to be activated simultaneously in CKD stage 3a and are preceded by elevated concentrations of total FGF23 likely due to increased cleavage of iFGF23.

## Disclosure

DH received speaker fees and research grants from Chiesi and Kyowa Kirin. IB received speaker fees and a travel grant by Kyowa Kirin. All the other authors declared no competing interests.

## Supplementary Material

Supplementary File (PDF) **Figure S1.** Blood hemoglobin, parameters of iron metabolism and bicarbonate in children with chronic kidney disease (CKD) patients as a function of estimated glomerular filtration rate (eGFR). **Table S1.** Medication for treatment of mineral and bone disorder in children with CKD stage 2 to 5D. **Table S2.** Parameters of renal calcium and phosphate excretion, iron metabolism and bicarbonate in children with CKD stages 2 to 5D.

## Footnotes

Supplementary File (PDF)
**Figure S1.** Blood hemoglobin, parameters of iron metabolism and bicarbonate in children with chronic kidney disease (CKD) patients as a function of estimated glomerular filtration rate (eGFR). **Table S1.** Medication for treatment of mineral and bone disorder in children with CKD stage 2 to 5D. **Table S2.** Parameters of renal calcium and phosphate excretion, iron metabolism and bicarbonate in children with CKD stages 2 to 5D.
